# Exercise-Induced Hematuria as the Main Manifestation of Migration of Intrauterine Contraceptive Device into the Bladder

**DOI:** 10.1155/2012/736426

**Published:** 2012-12-06

**Authors:** Michel Platiny Mascarenhas, Ricardo Brianezi Tiraboschi, Victor Pereira Paschoalin, Ellen Almeida Possidonio Costa, Carlos Henrique Suzuki Bellucci, José Bessa Junior

**Affiliations:** ^1^Division of Urology, School of Medicine, State University of Feira de Santana, Feira de Santana, Bahia 44031-460, BA, Brazil; ^2^Uroclínica of Joinville, Joinville, Santa Catarina 89201-700, SC, Brazil

## Abstract

Intrauterine device (IUD) is a common contraceptive method, due to its cost-effectiveness and low complication rates. Uterine perforation is a possible complication and IUD migration to the bladder is a rare and morbid condition. The present report describes an interesting case in which the urinary manifestations started 13 years after insertion, and the main clinical finding was exercise-induced hematuria.

## 1. Introduction

Intrauterine device (IUD) is a widely used reversible contraceptive method, due to its cost-effectiveness and low complication rates [1]. Serious complications are rare; however migration to adjacent pelvic organs is described [2], including the bladder with stone formation and lower urinary tract symptoms [3]. We describe a case of intravesical IUD migration with urologic manifestations started 13 years after insertion, and the main clinical finding was exercise-induced hematuria. 

## 2. Case Report

A previously healthy 39-year-old woman presented with recurrent episodes of gross hematuria after middle distance running (5 to 10 km) that ceased some hours after sports activity, rest, and hydration. Moreover, she reported dysuria, urge to urinate, and suprapubic pain, which also worsened with physical activity. Those symptoms were present for the past 4 months and worsened progressively, becoming more intense in recent weeks. In her previous medical and gynecologic anamnesis, she informed that 13 years ago she underwent implantation of IUD, few weeks after cesarean delivery. After the IUD implant, she complained of suprapubic pain and vaginal bleeding treated with rest and analgesics, with remission of symptoms after approximately 1 month. She was assessed by vaginal ultrasound and informed that IUD was not visualized; twelve years after the implantation of IUD she became pregnant again and had a second cesarean.

**Figure 1:**
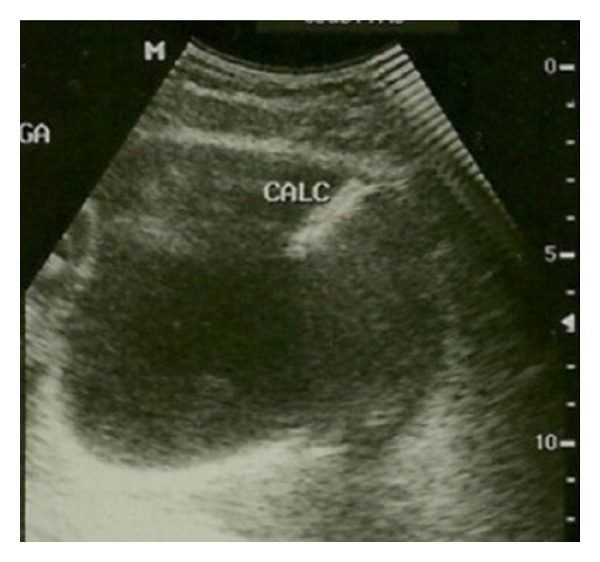
Transabdominal ultrasonography showing a 20 mm calculus located in the bladder dome.

**Figure 2:**
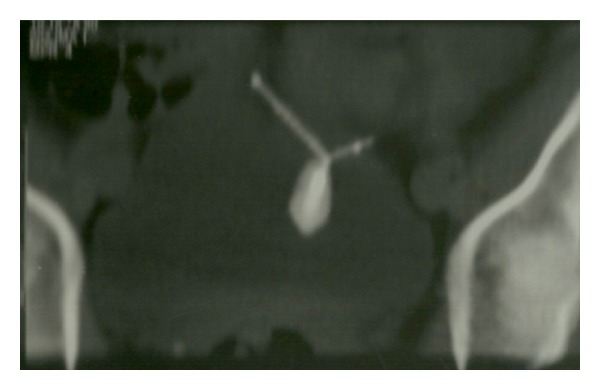
Pelvic helical computed tomography revealed a metallic foreign body partially located intravesically with adjacent calcification.

**Figure 3:**
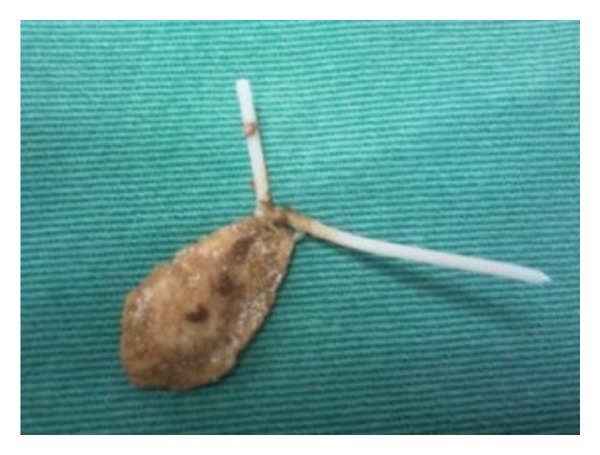
Intrauterine device extracted endoscopically, demonstrating partial stone formation.

Gynecologic examination was normal, urinalysis revealed microhematuria, and leukocyturia and urine culture demonstrated the presence of *Escherichia coli*. She was treated with antibiotics, with remission of symptoms only for 4 weeks. Due to recurrence of the symptoms, she was referred to the urologic department for further evaluation. Ultrasonography demonstrated a 20 mm calculus in the bladder dome (Figure 1) and pelvic helical computed tomography revealed a metallic foreign body partially located intravesically with adjacent calcification (Figure 2). Cystoscopy, performed under anesthesia, confirmed the intravesical IUD (partial), complicated by stone that was grasped by forceps, and extracted completely through the endoscopy (Figure 3). The patient was discharged one day after removing the IUD, with uneventful evolution.

## 3. Discussion

Although infrequent, one of the major complications of IUD is perforation of uterine wall with incidence ranging from 0.2 to 10 per 1000 insertions. Furthermore, intravesical migration with secondary bladder stone is yet more rare [3] and the exact mechanism that explains uterine perforation and IUD migrations is not entirely known [4]. In the present case, two other aspects can be highlighted: the long time (about 13 years) between the device implantation and the appearance of symptoms and exercise-induced hematuria as the primary clinical manifestation.

Among the factors related to uterine perforation, early insertion and former surgery are described [4]. In this case, the IUD was inserted a few weeks after cesarean. It's believed that uterine perforation may occur at the time of insertion or at any time thereafter. In the present case, pain and vaginal bleeding following implantation suggest that perforation may have occurred at the time of insertion. On the other hand, bladder erosion and intravesical foreign bodies are usually very symptomatic [2] and the initiation of urinary manifestations 13 years after IUD insertion suggests that intravesical migration may have occurred in the long term.

Exercise-induced hematuria has been described and becoming a common diagnosis in several sports, such as long distance running, football, soccer, boxing, and bike riding [5]. However, a number of genitourinary tract diseases, including bladder tumor, stones, or congenital hydronephrosis, may also cause gross or microscopic hematuria in some physically active patients. Despite the apparent kindness, exercise-induced hematuria should be considered a diagnosis of exclusion and further evaluation is recommended, even in recurrent hematuria, when other bladder symptoms occur or in patients with increased risk for malignancies [5, 6]. Ultrasonography is a sensitive diagnostic tool of bladder diseases and in cases of loss of the IUD. However, in cases of partial migrations, further diagnostic methods may be required. In this context, computerized tomography is very effective in demonstrating the IUD relations with adjacent structures and allows us to evaluate other possible causes of hematuria. Finally, cystoscopy is the optimal approach in evaluating intravesical calcifications and in the case of diagnosis already defined by imaging methods, endoscopy is performed with therapeutic intention.

Endoscopy extraction is the preferred approach in the removal of intravesical foreign bodies. Lithotripsy of bladder stones may be required preceding the extractions. In current days, open surgery is restricted to special conditions, for the removal of IUDs with formation of big stones or when technical resources are limited. Some cases were also treated with laparoscopy approach, a minimally invasive alternative to open surgery [4]. 

It should be remembered that patients with urinary symptoms and a history of retained IUD must be investigated for possible perforation of the uterus and intravesical migration of the device, that must be promptly removed [4, 7]. Endourological management is the preferred approach due to high success rate [7] and less morbidity; however open and videolaparoscopy surgeries may be required in challenging cases.
